# Aseptic loosening of tumor prostheses in distal femur after revision surgery: a retrospective study

**DOI:** 10.1186/s12957-023-03047-0

**Published:** 2023-05-31

**Authors:** Ziming Li, Xiuchun Yu, Ming Xu, Kai Zheng, Ziwei Hou, Zukang Miao, Yanshun Sun

**Affiliations:** 1 First Clinical Medical College, Shandong University of Traditional Chinese Medicine, Jinan, Shandong Province China; 2 Department of Orthopaedics, The 960Th Hospital of the PLA, Jinan, China; 3 School of Public Health, Weifang Medical College, Weifang, Shandong Province China

**Keywords:** Revision surgery, Distal femur, Tumor prostheses, Aseptic loosening, Retrospective study

## Abstract

**Background:**

Tumor prostheses of the distal femur after revision surgery is associated with high rates of aseptic loosening, which has introduced great challenges to the survival of patients, but only a few studies have evaluated their X-ray imaging. The purpose of this study was to analyze the risk factors for recurrence of aseptic loosening and make recommendations to reduce the incidence of aseptic loosening after revision surgery of tumor prostheses in the distal femur.

**Method:**

A retrospective analysis was performed on 23 patients who had revision surgery for distal femur prostheses due to aseptic loosening between June 2002 and June 2021. They were divided into two groups based on the condition of the prostheses after revision surgery: loosening group (9 patients) and control group (14 patients). Following the initial replacement, the length and diameter of the prosthetic intramedullary stem were measured through the standard full-length anteroposterior X-ray imaging of both lower limbs. The osteotomy length, femoral length and diameter, femoral intramedullary stem diameter, hip-knee-ankle angle (HKAA), mechanical lateral distal femoral angle (mLDFA), mechanical medial proximal tibial angle (mMPTA), and so on were measured as well. Following that, statistical analysis was performed.

**Results:**

Patients in the loosening group had statistically significant differences in the ratio of prostheses length to femur length (71.89 ± 6.62) and the ratio of intramedullary stem diameter to femoral diameter (25.50 ± 6.90) (*P* < 0.05), when compared to the control group. The HKAA (175.58 ± 2.78), mLDFA (94.42 ± 2.57), and the deviation angle between the lower limb alignment and the tibial prostheses force line (2.23 ± 1.09) in the loosening group were significantly different from those in the control group (*P* < 0.05) on postoperative radiographs of the entire length of the lower limbs. The lowest score in intramedullary manubrium I indicated less osteolysis, while the highest score in intramedullary manubrium III indicated the most serious osteolysis, and the difference was statistically significant (*P* < 0.05).

**Conclusions:**

Our study suggests that the use of longer and thicker intramedullary stems can effectively decrease the occurrence of aseptic loosening. Additionally, it is important to avoid using the original prostheses and reconstruct the standard line of lower limb force to further reduce the incidence of aseptic loosening. It is crucial to closely monitor the distal segment of the intramedullary stem for osteolysis after surgery.

## Introduction

Tumor segmental resection and prostheses replacement are a critical treatment method for limb bone tumors [1, 2]. Because bone tumors are more common in the distal femur [3–5], the design of tumor knee prostheses and tumor prostheses replacement in the distal femur are well developed. However, as patients’ survival times and life expectancies increase [6], the incidence of mechanical complications such as aseptic loosening and structural failure rises [7]. Henderson classified complications into five types [8], with aseptic loosening being the most common [1, 9]. More patients are having prostheses revision surgery because of aseptic loosening, but the revision failure rate is also increasing [10–13], which is mainly due to the recurrence of aseptic loosening [14]. Patients with aseptic loosening are often accompanied by increased weight-bearing pain, and radiographs show varying degrees of sinking, displacement, and the formation of surrounding clear bands. We also found these problems in the revision of distal femur prostheses. Some patients experienced second aseptic loosening after the first revision surgery and required second revision. Repeated revision surgery not only puts patients under a lot of physical, psychological, and financial stress but also it makes surgery more difficult because of anatomical position dislocation, thin bone, and soft tissue contracture caused by repeated surgery. According to literature studies, revision prostheses have more complications than initial replacement prostheses [15, 16]. As a result, reducing the incidence of secondary revision surgery and paying attention to the long-term effect of prostheses are a huge challenge for bone oncologists [17].

Aseptic loosening all occurred on the femur side in patients with distal femur bone tumors who underwent tumor segmental resection and tumor prostheses replacement, and no cases of aseptic loosening of tibial prostheses have been observed so far. The key structure connecting the prostheses to the femur is the intramedullary stem on the femoral side [18]. The stability of the tumor prostheses is affected by the connection between the intramedullary stem and the femur. Therefore, intraoperative adaptation of the intramedullary stem is critical to the success of revision surgery. Zhang et al. proposed to reduce the prosthesis failure rate by controlling the length of the medullary stem at around 143 mm [18]. Geiger et al. suggested that utilizing implants with the largest possible stems is important to revision cemented distal femoral replacements [19]. Piakong et al. suggested that a curved stem with a larger diameter could reduce the revision rate [20]. However, there is a few study as we know that answer questions as the following: how to choose the prosthetic intramedullary stem in revision surgery? Should the original intramedullary stem be used? Should a longer intramedullary stem be used? Or should a thicker intramedullary stem be used? This is an urgent clinical problem that must be addressed [21].

The lower limb alignment is the “gravitational line of the legs,” and the correct reconstruction of the limb alignment in total knee arthroplasty is an important factor affecting the quality of the surgery and the recovery of the knee function after surgery. The lower limb alignment is equally important in tumor prostheses replacement [22]. Periprosthetic osteolysis is still one of the major limitations of prosthetic longevity [23]. But there is no literature on osteolysis of tumor prostheses among the numerous osteolysis literatures.

In this study, we examined the patients’ X-ray films after the revision surgery and measured the length of the prosthetic stem, diameter of the prosthetic stem, osteolysis around the tumor prostheses and lower limb alignment, and so on, to finding the causes of aseptic loosening of the revision prostheses and make recommendations to reduce the incidence of aseptic loosening.

## Materials and methods

### Inclusion and exclusion criteria

**Fig. 1:**
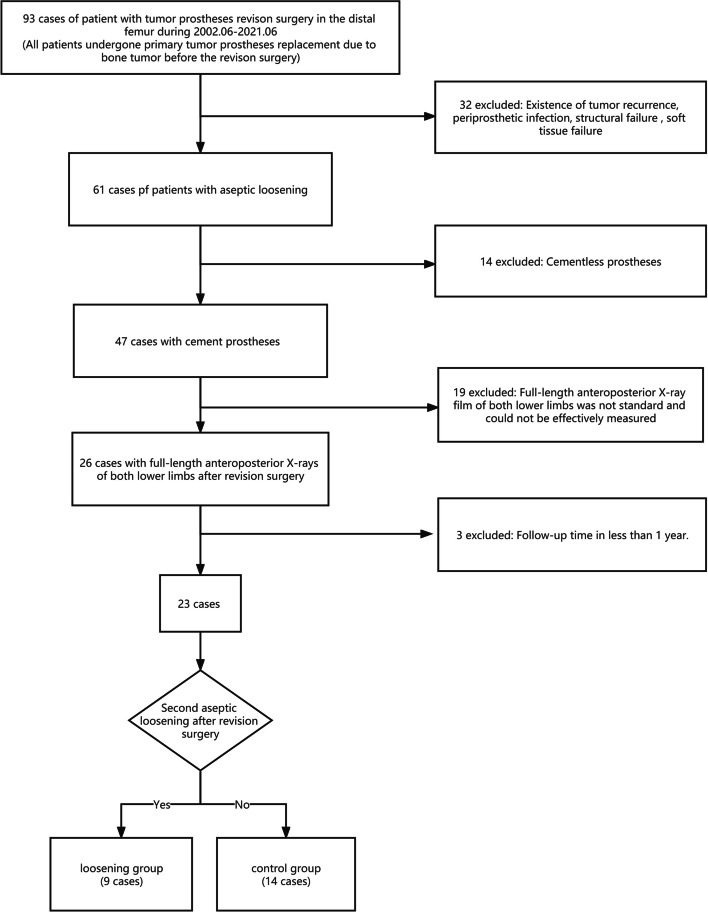
The flow diagram of included and excluded studies

The inclusion and exclusion criteria of the studies were shown in Fig. 1.

### Patients

A total of 23 patients who underwent revision surgery of distal femur prostheses due to aseptic loosening from June 2002 to June 2021 in PLA’s 960th Hospital were selected. The mean age of the patients was 44.39 ± 12.41 years old, including 15 males and 8 females, with a male-to-female ratio of 1.875:1. There were 13 cases of giant cell tumor of bone, 7 cases of osteosarcoma, 2 cases of malignant fibrous histiocytoma, and 1 case of chondrosarcoma. Seven patients underwent chemotherapy, and 7 patients had leg unequal length (affected limb shortening > 3 cm) before revision (Table 1).

**Table 1:** Demographic data of the patients

Case	Gender	Age of operation	Aseptic loosening occurred again after revision surgery	Time to revision (M)	Pathological diagnosis	Frequency of chemotherapy
1	M	38	Yes	28.77	Giant cell tumor of bone	0
2	M	59	Yes	344.13	Giant cell tumor of bone	0
3	M	42	Yes	75.13	Giant cell tumor of bone	0
4	M	52	Yes	133.61	Giant cell tumor of bone	0
5	M	58	Yes	32.27	Malignant fibrous histiocytoma	CVADIC-10
6	F	33	Yes	116.83	Giant cell tumor of bone	0
7	F	61	Yes	108.00	Giant cell tumor of bone	0
8	M	40	Yes	131.65	Giant cell tumor of bone	0
9	M	17	Yes	61.81	Osteosarcoma	DIA-9
10	M	51	No	174.20	Giant cell tumor of bone	0
11	M	56	No	335.00	Giant cell tumor of bone	0
12	M	45	No	9.67	Chondrosarcoma	0
13	F	64	No	238.97	Giant cell tumor of bone	0
14	M	40	No	132.06	Giant cell tumor of bone	0
15	F	42	No	93.87	Osteosarcoma	DIA-9
16	F	56	No	11.57	Malignant fibrous histiocytoma	0
17	M	49	No	119.79	Giant cell tumor of bone	0
18	M	31	No	156.83	Osteosarcoma	DIA-11
19	M	31	No	56.00	Osteosarcoma	DIA-10
20	F	25	No	95.77	Osteosarcoma	MMIA-6
21	M	37	No	168.32	Giant cell tumor of bone	0
22	F	56	No	79.40	Osteosarcoma	0
23	F	38	No	137.57	Osteosarcoma	DIA-12

At the end of the study, all 23 patients survived, including 1 patient who underwent knee arthrodesis 5 years after revision surgery due to aseptic loosening. Nine of the 23 patients had aseptic loosening again after revision surgery and were, therefore, included in the loosening group. The remaining 14 patients in this study had no complications such as aseptic loosening by the end of the follow-up period and were classified as control group.

### Prostheses

All of the knee prostheses were tumor type, with 15 customized prostheses (manufactured by Beijing Lidakang Company, China) and 8 combined prostheses (provided by Shandong Weigao Company, China). There were 2 fixed hinge knee prostheses and 21 rotary hinge knee prostheses. There were 2 curved stem cases and 21 straight stem cases. Bone cement was used to secure all prostheses.

### Surgical methods

The patient was placed in a supine position after a successful general anesthesia, and the surgical area was routinely disinfected, draped, and covered with a protective film. The original surgical incision was made and extended. The skin, subcutaneous tissue, and fascia layer were cut layer by layer, the scar tissue around the knee joint was cut, the joint capsule was cut medial to the patellar, the space between the vastus lateralis and the rectus femoris muscle was separated, the intermedius femoris muscle was split, and the femoral shaft and the artificial knee joint were exposed by pushing and stripping. After the dislocation of the knee prostheses, the femur end was lifted retrograde, the callus around the prostheses was chiseled out, and the femoral prostheses were removed. The intramedullary boundary membrane tissue and residual bone cement were thoroughly removed with curettage. After the tibia was treated, the tibia prostheses was retrogradely punched out in the medial foramen of the tibia nodule, and the bone cement in the tibia medullary cavity was further scraped. After the distal medullary cavity was expanded, the hydrogen hydroxide and normal saline were rinsed repeatedly, the medullary cavity and incision were rinsed with pulse pressure, and the bone cement was injected into the bone marrow cavity of the tibia and femur; new tibia prostheses and femur prostheses of appropriate length and thickness were inserted. Following the solidification of the bone cement, the knee prostheses were reset, the polyethylene meniscus pad was placed, and the flexion and extension mobile knee joints demonstrated good force lines and activities. After the hemostatic was completely removed, hydrogen peroxide and a large amount of normal saline were rinsed, patella was trimmed, the instruments and dressing were checked, the drainage tube was placed, the surgical incision was closed layer by layer, and sterile dressing was bound and fixed.

### Imaging evaluation

1. The length and diameter of the intramedullary stem of the prostheses after the initial replacement
2. Osteotomy length, femoral length, extramedullary length of femoral prostheses, intramedullary stem length of femoral prostheses, intramedullary stem diameter of femoral prostheses, femoral diameter, HKAA, mLDFA, mLDFA, the deviation angle between the lower limb alignment and the femoral prostheses force line, and the deviation angle between the lower limb alignment and the tibial prostheses force line after the revision surgery

The aforementioned measurements were made by three physicians in our department, and the data were averaged.

### Measurement method

The standard full-length anteroposterior X-ray of both lower limbs of the patient was measured using picture archiving and communication system (PACS, Qingdao Medicom Digital Engineering Company, China) with built-in length and angle. All patients stood on an X-ray large-plate multifunctional digital fluoroscopy system (Shimadzu-Hama Narayaki II, Japan) with the lower leg pressed against the plate, knees straight, feet together and flat on the weight-bearing plate, and both knees moderately internally rotated by about 10–15° so that the small head of the fibula overlapped the tibia by about one-third and the patella was oriented anteriorly. During measurement, the femoral head center (concentric circle method), the knee joint center (the midpoint of the intercondylar fossa of the femur and the tibial crest), and the ankle joint center (the midpoint of the line between the surface of the medial and lateral malleolus through the articular surface of the distal tibia) were marked.

### Statistical analysis

Statistical analysis was performed using SPSS 25.0 (IBM Corporation, USA) statistical software. Normally distributed measurement data were expressed as mean ± standard deviation ($\overline{x }$ ± S) and analyzed using the *T*-test on two independent samples; non-normally distributed measurement data were analyzed using the Kruskal–Wallis multiple independent samples method. Fisher’s chi-square test was used to analyze the count data. Binary logistic regression analysis was performed for risk factors, and odds ratio values and 95% confidence intervals were calculated.

## Results

### General result

Five patients (55.56%) in the loosening group had unequal lower limb length, which was statistically significant difference compared to 2 patients (14.29%) in the control group (*P* < 0.05).

There was no significant difference in age between the loosening group (44.44 ± 14.49) years and the control group (44.36 ± 11.47) years (*P* > 0.05). Time interval between primary replacement and primary revision in the loosening group was 114.69 ± 94.79 months, which was not significantly different from that in the control group 129.22 ± 86.48 months (*P* > 0.05). Two patients in the loosening group (22.22%) and 5 patients (35.71%) in the control group received chemotherapy, which did not show statistically significant difference (*P* > 0.05) between two groups (Table 2).

**Table 2:** Comparison of general data

	Loosening group	Control group	Χ^2^/T	*P*
Age [years, ($\overline{x }$ ± S)]	44.44 ± 14.49	44.36 ± 11.47	0.016	0.987
Time interval between primary replacement and primary revision [months, ($\overline{x }$ ± S)]	114.69 ± 94.79	129.22 ± 86.48	− 0.756	0.477
Existence of unequal length of lower limbs [number of cases, (%)]	5 (55.56)	2 (14.29)	19.398	0.001
Receiving chemotherapy [number of cases, (%)]	2 (22.22)	5 (35.71)	0.471	0.657

### Risk factors for recurrence of aseptic loosening

Compared with control group, patients in loosening group had statistical differences in the ratio of prostheses length to femur length (71.89 ± 6.62) and the ratio of intramedullary stem diameter to femoral diameter (25.50 ± 6.90) (*P* < 0.05). The osteotomy ratio and the length ratio of the extramedullary and intramedullary parts of the femoral prostheses did not differ statistically.

In terms of the increase proportion of length and diameter of the intramedullary stem during the initial replacement and prostheses revision, the increase proportion of loosening length (3.61 ± 7.23) and diameter (3.95 ± 4.59) were not statistically significant difference compared with the control group (*P* > 0.05) (Table 3).

**Table 3:** Comparison of prostheses measurements between the loosening group and control group

	Loosening group	Control group	T/Z	*P*
Osteotomy ratio [%, ($\overline{x }$ ± S)]	46.06 ± 0.11	44.24 ± 28.08	0.307	0.762
The length ratio of the extramedullary and intramedullary parts of the femoral prostheses [%, ($\overline{x }$ ± S)]	96.27 ± 28.08	108.51 ± 74.38	− 0.469	0.644
The ratio of prostheses length to femur length [%, ($\overline{x }$ ± S)]	71.89 ± 6.62	80.75 ± 8.39	− 2.672	0.014
The ratio of intramedullary stem diameter to femoral diameter [%, ($\overline{x }$ ± S)]	25.50 ± 6.90	35.06 ± 12.18	− 2.457	0.014
Growth ratio of intramedullary stem length^a^ [%, ($\overline{x }$ ± S)]	3.61 ± 7.23^b^	10.45 ± 21.23^b^	− 0.615	0.551
Growth ratio of intramedullary stem diameter [%, ($\overline{x }$ ± S)]	3.95 ± 4.59^b^	28.78 ± 26.71^b^	− 1.804	0.099

^a^Growth ratio = (length of prostheses after revision — length of prostheses after initial replacement)/length of prostheses after initial replacement

^b^The original prostheses were reimplanted in 4 patients, so the growth rate was 0, which increased the dispersion of the data, but it conformed to the normal distribution

The HKAA (175.58 ± 2.78), mLDFA (94.42 ± 2.57), and the deviation angle between the lower limb alignment and the tibial prostheses force line (2.23 ± 1.09) in the loosening group were significantly different from those in the control group (*P* < 0.05), according to postoperative X-ray films of the full length of the lower limbs (Table 4).

**Table 4:** Comparison of lower limb alignment between the loosening group and control group

	Loosening group	Control group	T	*P*
HKAA [°, ($\overline{x }$ ± S)]	175.5 ± 2.78	177.50 ± 1.25	− 2.268	0.034
mLDFA [°, ($\overline{x }$ ± S)]	94.42 ± 2.57	92.76 ± 1.24	2.129	0.045
mMPTA [°, ($\overline{x }$ ± S)]	91.50 ± 1.42	91.14 ± 0.78	0.777	0.446
The deviation angle between the lower limb alignment and the femoral prostheses force line [°, ($\overline{x }$ ± S)]	5.88 ± 1.90	6.75 ± 1.78	− 1.109	0.280
The deviation angle between the lower limb alignment and the tibial prostheses force line [°, ($\overline{x }$ ± S)]	2.23 ± 1.09	0.83 ± 0.54	3.596	0.004

Binary logistic regression analysis was performed on the following single factors. We found that the ratio of extramedullary length to the intramedullary stem length greater than 1, the ratio of the length of the prostheses to the length of the femur less than 0.8, the ratio of intramedullary stem diameter to the femoral diameter less than 30%, and the existence of genu valgus and genu varus after surgery were not risk factors for aseptic loosening of prostheses (Table 5).

**Table 5:** Logistic regression analysis of risk factors affecting prostheses aseptic loosening

Variable	*OR* ratio	95% *CI*	*p*-value
The ratio of extramedullary length to the intramedullary stem length greater than 1	0.800	0.149, 4.297	0.795
The ratio of the length of the prostheses to the length of the femur less than 0.8	4.467	0.702, 31.036	0.111
The ratio of intramedullary stem diameter to the femoral diameter less than 30%	0.167	0.016, 1.718	0.132
Knee valgus or genu varus after surgery^a^	5.000	0.821, 30.461	0.081

^a^Knee varus was defined as a postoperative hip-knee-ankle angle < 177°, and genu valgus was defined as a postoperative hip-knee-ankle angle > 183

### Osteolysis around the intramedullary stem during the progression of aseptic loosening

**Fig. 2:**
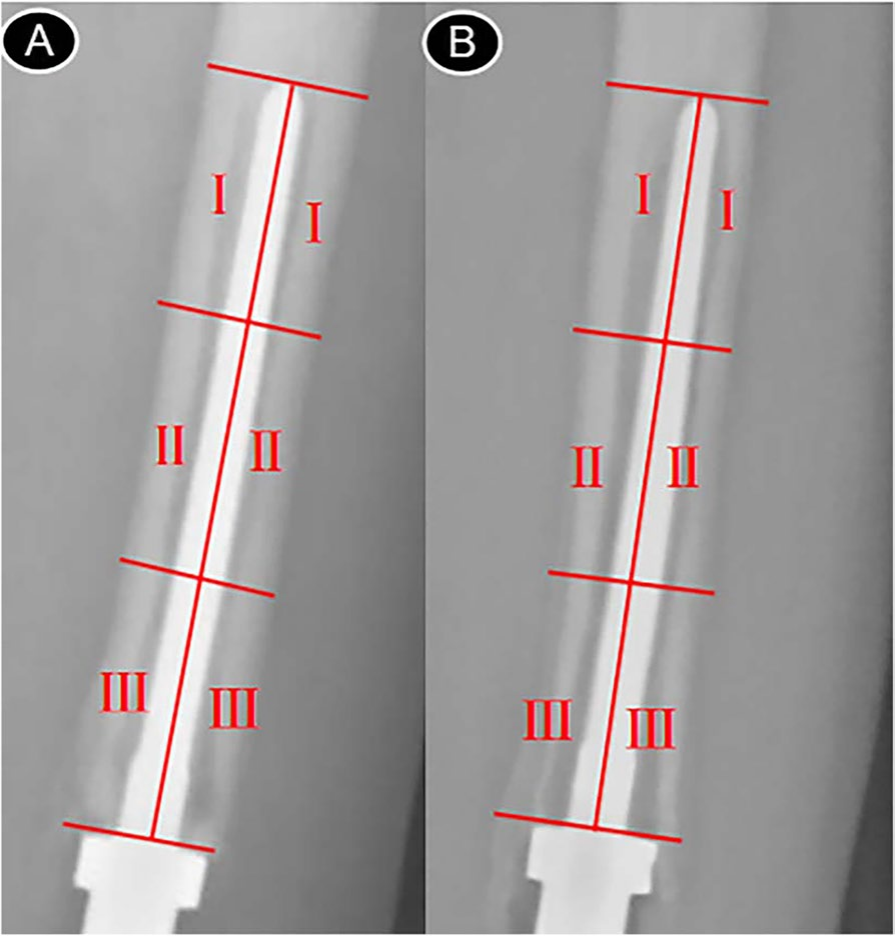
**A** and **B** are X-rays of the same patient at different times. **A** shows partition of the intramedullary stem after underwent tumor prostheses replacement. **B** shows partition of the intramedullary stem before revision surgery

Local or intraosseous bone resorption was defined as osteolysis [24]. We analyzed imaging on X-ray films of the femoral intramedullary stem in 13 patients undergoing prosthetic revision during the progression of aseptic loosening, with 8 groups after replacement to before revision surgery and 5 groups after first to second revision surgery. The X-ray films in the same group were set to the same ratio and gray scale, and the intramedullary stem was divided into six equal areas including two I, II, and III each along the prostheses longitudinal axis (Fig. 2). Osteolysis score was defined as 1 score in the lateral cortex and 1 score in the medial bone. During the progression of aseptic loosening, bone changes in various regions of the intramedullary stem were observed. X-ray films revealed that all cases had visible bone loss around the prostheses, in some cases cancellous osteolysis, and in others cortical osteolysis. Table 6 showed the scores of 13 patients, and the scores were tested using a nonparametric test.

**Table 6:** Kruskal–Wallis *H*-test for the scores of osteolysis areas I, II, and III

	I	II	III
Osteolysis score	15.38	18.19	26.42
Kruskal–Wallis *H*-test	6.971		
*P*	0.031		

In this study, it was found that the score in area I of the intramedullary stem was the lowest, indicating less osteolysis, and the score in area III of the intramedullary stem was the highest, indicating the most severe osteolysis, and the difference was statistically significant (*P* < 0.05).

## Discussions

### The importance of revision surgery

After revision surgery for tumor prostheses, aseptic loosening occurs again. Revision surgery should be actively performed without significant tumor factors affecting patients’ life and health. According to Heyberger’s study, prostheses revision surgery could enable patients to achieve similar joint function to the initial replacement, while disease-specific and health-related outcomes were improved [25]. Despite the fact that revision surgery of tumor prostheses is difficult and has a high failure rate [15], it remains the best option for patients seeking to save limbs and improve limb function [13]. Patients can still have good lower limb function after revision, which helps to improve their quality of life.

### Risk factors leading to aseptic loosening after revision surgery

Aseptic loosening is a multifactorial interaction involving both mechanical (prostheses wear, fretting, stress shielding, structural design, etc.) and biological factors (chronic inflammatory response, osteolysis-related cytokine release, and enzyme activation). Chemotherapy had no effect on the aseptic loosening of the prostheses, according to the findings of this study. Most studies assumed that chemotherapy would inhibit the growth of biologically fixed bone and affect the prostheses stability [26]. Pugh’s study, on the other hand, concluded that the incidence of aseptic loosening of bone cement prostheses was low regardless of whether chemotherapy was administered [27]. As a result, we believed that chemotherapy will not cause cemented prostheses loosening.

Aseptic loosening is affected by prostheses length and diameter. Longer femur prostheses, particularly intramedullary stems, provide more bone-cement-prostheses contact area, which improves stability. Shorter femur prostheses has shorter arms. The ratio of extramedullary part to intramedullary of the prostheses was 96.27% ± 28.08%, which was not statistically different from that of 108.51% ± 74.38% in the loosening group. We believed this was due to the higher osteotomy ratio in revision surgery. A larger proportion could result in a longer moment arm, and the stability of the prostheses was dependent on the extension of the moment arm’s distal fixation, as well as the insertion and locking of the internal wall of the femoral bone marrow cavity. Longer prosthetic lengths could be obtained by lengthening the intramedullary stem and reducing the length of the extramedullary part. Bergin et al. discovered that a larger intramedullary stem/femoral diameter ratio could reduce the rate of prostheses loosening [28], and our study found a similar result, with a diameter of 35.06% ± 12.18% in the control group and 25.50% ± 6.90% in the loosening group. The larger diameter of the intramedullary stem allowed it to completely fill the femoral medullary cavity, and a good press fit reduced prostheses fretting. Simultaneously, the larger diameter of the intramedullary stem reduced the amount of bone cement used and the rate at which it dissolves. Bone cement has poor torsion resistance, is non-degradable, and has no osteoinductive and conductive capabilities. The literature suggested that bone cement wear debris may activate T cells and macrophages around the prostheses, as well as osteoclasts, causing osteolysis [29].

In tumor prosthetic revision surgery, the lower extremity line of alignment is critical. Our study confirmed this view, with a HKAA of 177.50° ± 1.25° in the control group and 175.5° ± 2.78° in the loosening group. At the apex of the intramedullary stem, the deviation of the anatomical axis and the line of force is very large, as is the resulting bending moment. The deviation of lower limb alignment will increase the shear stress on the prostheses and cause uneven distribution of conduction. Rubbing the apex of the intramedullary stem against the femur increased the possibility of the prostheses protruding through the cortex and causing aseptic loosening. The deviation of lower limb alignment and aseptic loosening are mutually influencing processes. Aseptic loosening of the prostheses will obviously aggravate gravity line deviation, and the deviation of lower limb alignment will aggravate friction between the prostheses and the femur, aggravate the fretting of the prostheses and the formation of wear particles, and accelerate the occurrence of aseptic loosening. This is also why aseptic loosening is more common in patients with lower limb shortening. The primary cause of shortened limbs is osteolysis caused by aseptic loosening, which causes the prostheses to settle and shift.

### Femoral osteolysis during the progression of aseptic loosening

Periprosthetic osteolysis is required for aseptic loosening [30], but mechanical stress/strain is also required to cause movement of the prostheses. A variety of factors contributed to aseptic loosening, including stress occlusion, stress concentration, prostheses fretting at the prostheses-bone cement interface, and wear particles. The main manifestations were the formation of a clear zone around the intramedullary stem due to a large number of osteolysis. For the study of bone changes, 13 groups of intramedullary peristem X-ray films were chosen after replacement and before revision. We found that osteolytic gaps at the prostheses-cement interface in many sets of X-ray films, which widened and expanded with the progression of aseptic loosening, eventually lead to implant loosening. Osteolysis was found less frequently near the apex of the intramedullary stem, more frequently at the distal end of the intramedullary stem, and only in the distal segment of the intramedullary stalk did osteolysis in the lateral cortex occur. Because osteolysis is triggered by T cells activated by bone cement, it is more likely to occur in bone cement-filled areas. During long-term wear, bone cement produces particles and debris, which can cause inflammation and osteoclast activation [31], resulting in osteolysis, which can be linear, i.e., evenly distributed around the prostheses, or localized, i.e., forming islands of bone loss closely associated with the prostheses. Both linear and localized osteolysis will aggravate the prostheses micro-movement, leading to aseptic loosening.

In this study, all prostheses were secured using bone cement which provided immediate stability and functionality during the early and middle stages. However, it was observed that bone cement had a tendency to become brittle due to fatigue and had a higher incidence of aseptic loosening caused by osteolysis during the later stages. This has been reported to be around 30% within 10 years [32, 33]. While cementless prostheses may have inferior short-term function compared to cement prostheses, they ultimately achieve superior stability once bone ingrowth and osseointegration are achieved [34]. The initial stability of cementless prostheses is crucial [35, 36]. If initial stability is not achieved, fretting can lead to the formation of fibrous tissue and aseptic loosening. Therefore, it is important to ensure sufficient intraoperative compression and postoperative support fixation for cementless prostheses to achieve initial postoperative stability.

### How to reduce the rate of aseptic loosening after revision surgery

Patients’ survival rates and survival times have been significantly improved with adjuvant support such as neoadjuvant chemotherapy and targeted tumor therapy [37]. Meanwhile, patients have increasingly high expectations for postoperative limb function, which places high demands on the service life of prostheses. It is reflected not only in the prostheses design, materials, processing technology, and so on but also in the surgeon’s operation skills.

When revising cemented distal femur prostheses, we recommend using longer and thicker intramedullary stems. In theory, an intramedullary stem of sufficient length and diameter can completely fill the medullary cavity, fully press fit, and reduce prosthetic fretting. It is recommended to use the longest and thickest intramedullary stem possible while preserving sufficient cortical bone, as well as an intramedullary stem that fits the anatomical structure of the femoral medullary cavity, such as a curved stem. We were opposed to using the original prostheses in revision surgery. This study included 4 patients who used the original prostheses, 3 of whom developed aseptic loosening again after revision surgery. Meanwhile, a study on the increase ratio of length and diameter of the intramedullary stalk revealed that a higher increase ratio was beneficial to the prostheses stability.

The application of modern bone cement filling techniques can help to reduce the rate of prostheses loosening after surgery [38].

When the prostheses is inserted, proper alignment of the lower limb must be restored. Correct reconstruction of lower limb alignment is a critical step for long-term prostheses stability and patient limb function recovery [22, 39]. The deviation of the anatomical axis and the lower limb alignment were significant at the tip of the intramedullary stem, as was the resulting bending moment. The deviation of lower limb alignment would increase and unevenly distribute stress on the prostheses, resulting in stress concentration and stress shielding, which would further friction the apex of the intramedullary stem with the femur and aggravate prosthetic fretting, leading to aseptic loosening. As a result, positioning the prostheses along the normal lower limb alignment is critical to the success of revision surgery.

## Conclusions

X-ray films of patients with aseptic loosening after revision surgery showed that the ratios of prostheses length to femur length and intramedullary stem diameter to femoral diameter were smaller than those of patients without loosening. Meanwhile, patients with aseptic loosening had different deviations of lower limb alignment than patients without loosening, but these were not risk factors for the recurrence of aseptic loosening. However, we continued to believe that using longer and thicker intramedullary stems effectively reduced the incidence of aseptic loosening, and we are against using the original prostheses for reconstruction. Reconstructing the standard line of lower limb force is also important to reduce the incidence of aseptic loosening. The distal segment of the intramedullary stem was more prone to osteolysis and should be monitored closely after surgery.

## Data Availability

The dataset supporting the conclusions of this article is available on request — please contact the corresponding author.

## Abbreviations

HKAA: Hip-knee-ankle angle
mLDFA: Mechanical lateral distal femoral angle
mMPTA: Mechanical medial proximal tibial angle
